# Modeling Chemotaxis Reveals the Role of Reversed Phosphotransfer and a Bi-Functional Kinase-Phosphatase

**DOI:** 10.1371/journal.pcbi.1000896

**Published:** 2010-08-19

**Authors:** Marcus J. Tindall, Steven L. Porter, Philip K. Maini, Judith P. Armitage

**Affiliations:** 1 Centre for Mathematical Biology, Mathematical Institute, University of Oxford, Oxford, United Kingdom; 2 Institute of Cardiovascular and Metabolic Research, School of Biological Sciences, University of Reading, Reading, United Kingdom; 3 Department of Mathematics, University of Reading, Reading, United Kingdom; 4 Oxford Centre for Integrative Systems Biology, Department of Biochemistry, University of Oxford, Oxford, United Kingdom; 5 School of Biosciences, University of Exeter, Exeter, United Kingdom; University of Illinois at Urbana-Champaign, United States of America

## Abstract

Understanding how multiple signals are integrated in living cells to produce a balanced response is a major challenge in biology. Two-component signal transduction pathways, such as bacterial chemotaxis, comprise histidine protein kinases (HPKs) and response regulators (RRs). These are used to sense and respond to changes in the environment. *Rhodobacter sphaeroides* has a complex chemosensory network with two signaling clusters, each containing a HPK, CheA. Here we demonstrate, using a mathematical model, how the outputs of the two signaling clusters may be integrated. We use our mathematical model supported by experimental data to predict that: (1) the main RR controlling flagellar rotation, CheY_6_, aided by its specific phosphatase, the bifunctional kinase CheA_3_, acts as a phosphate sink for the other RRs; and (2) a phosphorelay pathway involving CheB_2_ connects the cytoplasmic cluster kinase CheA_3_ with the polar localised kinase CheA_2_, and allows CheA_3_-P to phosphorylate non-cognate chemotaxis RRs. These two mechanisms enable the bifunctional kinase/phosphatase activity of CheA_3_ to integrate and tune the sensory output of each signaling cluster to produce a balanced response. The signal integration mechanisms identified here may be widely used by other bacteria, since like *R. sphaeroides*, over 50% of chemotactic bacteria have multiple *cheA* homologues and need to integrate signals from different sources.

## Author Summary

Chemotactic bacteria sense nutrient gradients and swim towards better environments for growth. A cluster of receptors in the cell membrane detects nutrient levels and signals via a cytoplasmic signaling pathway to the flagellum. The complexity of this signaling pathway varies across different bacterial species. The relatively simple pathway used by *Escherichia coli* is well understood; however, many bacteria, for example *Rhodobacter sphaeroides*, have more sophisticated pathways that, as well as being able to detect nutrients, are also able to assess the metabolic state of the cell. The receptors that detect metabolic state are located within an additional cluster that is physically distinct from the one that senses nutrients. In this work, we use a combination of experimentation and mathematical modeling to gain insight into the complex decision-making mechanisms that enable bacteria to weigh-up different stimuli and decide upon an appropriate response. We find novel communication mechanisms between the two signaling clusters that allow the outputs of the signaling pathways to be balanced and tuned according to the prevailing environmental conditions. The signaling principles identified here are likely to be used in other complex sensory networks.

## Introduction

Two-component signaling pathways are the major mechanism by which bacterial cells sense and respond to changes in their environment. They regulate processes as diverse as virulence, gene expression, development and motility [1]. Bacteria can have over 100 different two-component pathways per cell, one form of which controls swimming behavior. This chemosensory pathway has been extensively studied as an example of a two-component signaling pathway as it provides a model of signaling, signal termination and receptor adaptation. Mathematical modeling has proved particularly useful in helping to understand the complexity of *Escherichia coli* chemotaxis [2]–[10].

Most chemotactic bacteria sense changes in their extracellular environment using transmembrane chemoreceptors [11]. These chemoreceptors signal via an intracellular signaling cascade to the flagellar motor. In the case of *E. coli*, the signaling cascade is well understood [12], [13]. The chemoreceptors form a quaternary complex at the cell poles with the scaffold protein CheW and the histidine protein kinase, CheA [14]–[16]. The chemoreceptors detect changes in the periplasmic chemoeffector concentration and control the rate at which CheA autophosphorylates on a conserved histidine residue. In response to decreased attractant concentration, the chemoreceptors signal to increase the rate of CheA autophosphorylation [17]–[19]. Following autophosphorylation, the phosphoryl group is transferred from the histidine residue of CheA to an aspartate residue in one of the two response regulators (RRs), CheY or CheB [20]–[22]. CheY-P is released from the chemotaxis cluster and diffuses through the cell to the flagellar motor. CheY-P binds the FliM component of the flagellar motors, causing the direction of flagellar rotation to switch from counter-clockwise to clockwise resulting in tumbling of the bacterium [23], [24]. CheA-P also phosphorylates the methylesterase CheB, which facilitates adaptation of the chemoreceptor cluster [25], [26]. CheY-P and CheB-P both naturally autodephosphorylate [27], although the rate of CheY-P dephosphorylation is enhanced by CheZ to allow signal termination within the time required for effective gradient sensing [28], [29].

In contrast to *E. coli*, *Rhodobacter sphaeroides* has a more complex signaling pathway with multiple copies of the signaling proteins encoded by three major chemosensory operons [30]. Many other bacterial species appear to have multiple chemosensory operons as analysis of sequenced genomes suggests that ∼50% of species with any *che* genes have at least two *cheA*s [30]–[32]. This raises the question of how behavior is controlled by two or more homologous pathways and how sensory data from each of the pathways are integrated to produce a balanced response. Under laboratory conditions, *R. sphaeroides* swims using a single sub-polar unidirectional flagellum (Fla1), which is controlled by the protein products of *che*Op_2_ and *che*Op_3_ [33]–[38]. The intracellular signaling cascade controlling the Fla1 flagellum comprises three CheA kinase proteins (denoted CheA_2_, CheA_3_, CheA_4_), three CheY proteins (CheY_3_, CheY_4_ and CheY_6_) and two CheBs (CheB_1_, CheB_2_) [34], [35], [39]–[42]. CheA_3_ and CheA_4_ are unusual CheAs in that they lack some of the domains found in *E. coli* CheA and neither protein is capable of autophosphorylation [43]. However, together CheA_3_ and CheA_4_ have all of the activities of a functional CheA with CheA_4_ forming a homodimer that binds ATP and phosphorylates the Hpt domain of CheA_3_.

**Figure 1:**
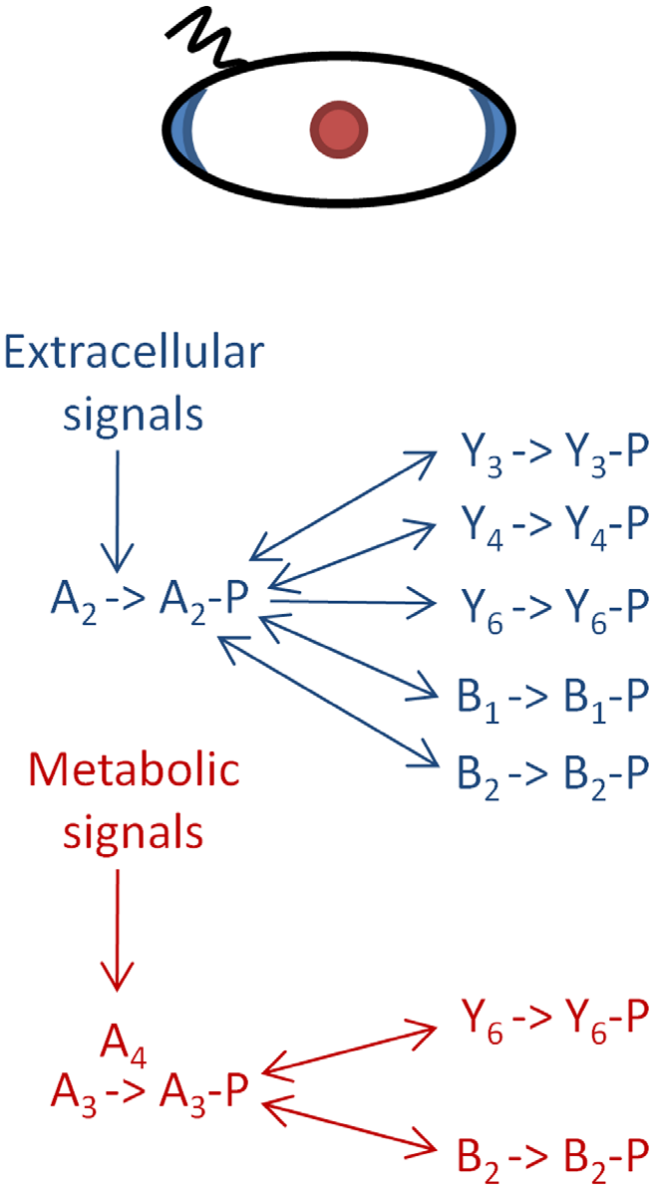
Diagram showing the RRs phosphorylated by the polar (blue) and cytoplasmic (red) chemotaxis clusters. The polar chemotaxis cluster contains CheA_2_ and responds to the external environment. CheA_2_ autophosphorylates and can then serve as a phosphodonor for all of the RRs. The cytoplasmic chemotaxis cluster contains CheA_3_ and CheA_4_, and is thought to respond to the metabolic state of the cell. CheA_3_ is phosphorylated by CheA_4_. Unlike CheA_2_-P, CheA_3_-P is not able to phosphorylate all of the RRs – it can only phosphorylate CheY_6_ and CheB_2_. All of the RR-Ps spontaneously autodephosphorylate; however, the dephosphorylation of CheY_6_-P is accelerated by the phosphatase activity of CheA_3_.

The signal transduction proteins are organized and localised into two distinct sensory clusters and the signaling output of both clusters is required for chemotaxis [43], [44]. CheA_2_ is located in a chemotaxis cluster at the cell poles, which comprises transmembrane chemoreceptors and the signal transduction proteins encoded by *che*Op_2_ [44]. This cluster detects changes in the periplasmic concentration of chemoeffectors. Previous data show that CheA_2_-P rapidly phosphorylates CheY_3_, CheY_4_, CheY_6_, CheB_1_ and CheB_2_ (Figure 1), although the kinetics of phosphotransfer differ in each case [45]. CheA_3_ and CheA_4_ localize to a second chemotaxis cluster found in the cytoplasm [44]. This cluster contains the signal transduction proteins encoded by *che*Op_3_ along with the soluble chemoreceptors and is believed to sense the metabolic state of the cell [44], [46]. CheA_3_-P rapidly phosphorylates only the RRs, CheY_6_ and CheB_2_ [43], [47]. In addition, CheA_3_ has an aspartyl-phosphate phosphatase activity that is specific for CheY_6_-P; this activity is required for the rapid signal termination that is necessary for chemotactic responses [48]. CheA_3_ in conjunction with CheA_4_ can therefore be considered to be a bifunctional kinase/phosphatase.

*In vitro* studies have shown that all of the *R. sphaeroides* CheYs can bind the flagellar switch protein, FliM, and that this binding is strongest when the CheYs are phosphorylated [49], but less is known about the effect of CheY/CheY-P binding to FliM on flagellar rotation. CheY_6_ is essential for chemotaxis and CheY_6_-P alone is capable of causing the chemotactic stop that is necessary for changing swimming direction [39]. However, CheY_6_ alone cannot support chemotaxis; either CheY_3_ or CheY_4_ are also required. Furthermore, phosphorylation site mutants of CheY_3_, CheY_4_ and CheY_6_ do not support chemotaxis [39], suggesting that phosphorylation of all of these CheYs is necessary for chemotaxis.

Thus there are two complete chemosensory pathways in *R. sphaeroides*, localized to different regions of the cell and with different patterns and kinetics of phosphotransfer to the RRs. However, the outputs of these two signaling pathways must be integrated to control the behavior of a single flagellar motor. *In vitro* biochemistry identified which RRs are phosphorylated by each CheA and the kinetics of the interactions, however, assessing the relative contribution made to RR-P levels by each of these CheAs *in vivo* is more complex, since all of the RR-Ps will be competing with one another for phosphorylation by the CheAs. We used mathematical modeling to predict the possible signaling pathways within this complex system and tested these predictions experimentally.

The aim of this study was therefore to combine our knowledge of the kinetic preferences of the signaling reactions gained from *in vitro* biochemistry with the *in vivo* data on protein copy number within a mathematical model that can predict the changes in RR-P levels resulting from changes in CheA activity at either cluster. This model was then used to analyze the contribution made by each cluster in controlling RR-P levels and the dynamics of the signaling reactions. Using the model, we identified unexpected key roles for reversed phosphotransfer between RR-P and CheA in the network, which would enable communication between the two sensory clusters and thus regulate the output signals. In addition, we demonstrated that the principal RR, CheY_6_, with the aid of its specific phosphatase, the bifunctional CheA_3_/CheA_4_ kinase, could act as a phosphate sink for the other RR-Ps. Regulation of the output of sensory networks by the activity of key kinase/phosphatase proteins is likely to be a common mechanism, but this is one of the first to be identified that balances the outputs of two interconnected pathways.

## Results

### Construction of the mathematical model

Within an *R. sphaeroides* cell, CheA_2_ has been shown to localize to the polar chemotaxis cluster, while CheA_3_ and CheA_4_ localize to the cytoplasmic cluster [44]. All the *R. sphaeroides* CheYs are free to diffuse throughout the cytoplasm of the cell enabling communication between the receptor clusters and flagellar motor. Unlike *E. coli*, the CheBs are also diffuse in the cytoplasm [39], [44]. As illustrated in Figure 1, CheA_2_-P can phosphorylate all of the RRs, whilst CheA_3_-P is only able to phosphorylate CheY_6_ and CheB_2_. What is the reason for this discrimination and how does it contribute towards the chemotactic response of the cell? To understand the role of each signaling cluster we constructed an ordinary differential equation (ODE) model of an *R. sphaeroides* cell as detailed in Text S1. The model integrates *in vivo* protein expression levels with *in vitro* data on the kinetic preference of the CheAs for each of the RRs to predict RR-P levels throughout a simulated chemotactic response. The model includes the phosphorylation reactions shown in Table 1 and was parameterized with published reaction rate constants and protein expression levels (Table 2).

**Table 1:** The phosphorylation reactions included in the model of the *R. sphaeroides* chemotaxis signalling pathway.

Reaction number	Reaction	Type
(1)	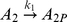	Autophosphorylation
(2)	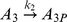	Phosphorylation by CheA_4_
(3)	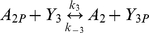	Phosphotransfer
(4)	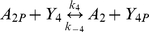	Phosphotransfer
(5)	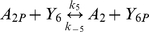	Phosphotransfer
(6)	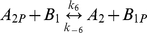	Phosphotransfer
(7)	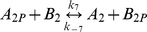	Phosphotransfer
(8)	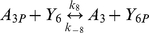	Phosphotransfer
(9)	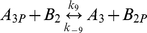	Phosphotransfer
(10)	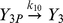	Autodephosphorylation
(11)	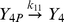	Autodephosphorylation
(12)	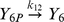	Autodephosphorylation
(13)	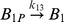	Autodephosphorylation
(14)	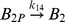	Autodephosphorylation
(15a)	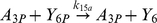	Phosphatase assisted dephosphorylation
(15b)	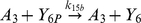	Phosphatase assisted dephosphorylation

**Table 2:** Parameter values directly determined from experimental data.

Rate	Description	Value	Standard error	Units	Source
*k_1_*	CheA_2_ autophosphorylation	0.12	0.02	s^−1^	[45]
*k_2_*	Phosphorylation of CheA_3_ by CheA_4_	0.98	0.17	s^−1^	[43]
*k_10_*	CheY_3P_ autodephosphorylation	1.93×10^−2^	0.20×10^−2^	s^−1^	[45]
*k_11_*	CheY_4P_ autodephosphorylation	1.82×10^−2^	0.13×10^−2^	s^−1^	[45]
*k_12_*	CheY_6P_ autodephosphorylation	1.69×10^−1^	0.12×10^−1^	s^−1^	[45]
*k_13_*	CheB_1P_ autodephosphorylation	1.73×10^−4^	0.06×10^−4^	s^−1^	[45]
*k_14_*	CheB_2P_ autodephosphorylation	1.33×10^−2^	0.12×10^−2^	s^−1^	[45]
*k_15a_*	CheY_6P_ dephosphorylation by CheA_3_	5.20×10^3^	0.32×10^3^	(Ms)^−1^	[48]
*k_15b_*	CheY_6P_ dephosphorylation by CheA_3P_	5.20×10^3^	0.32×10^3^	(Ms)^−1^	[48]
*A_2T_*	Total concentration of CheA_2_	89.9	7.6	µM	[69]
*A_3T_*	Total concentration of CheA_3_	89.9	10.4	µM	[69]
*Y_3T_*	Total concentration of CheY_3_	3.5	1.0	µM	[68]
*Y_4T_*	Total concentration of CheY_4_	13.8	2.8	µM	[68]
*Y_6T_*	Total concentration of CheY_6_	225	27	µM	[68]
*B_1T_*	Total concentration of CheB_1_	81.2	3.8	µM	[69]
*B_2T_*	Total concentration of CheB_2_	20.8	2.1	µM	[69]

**Figure 2:**
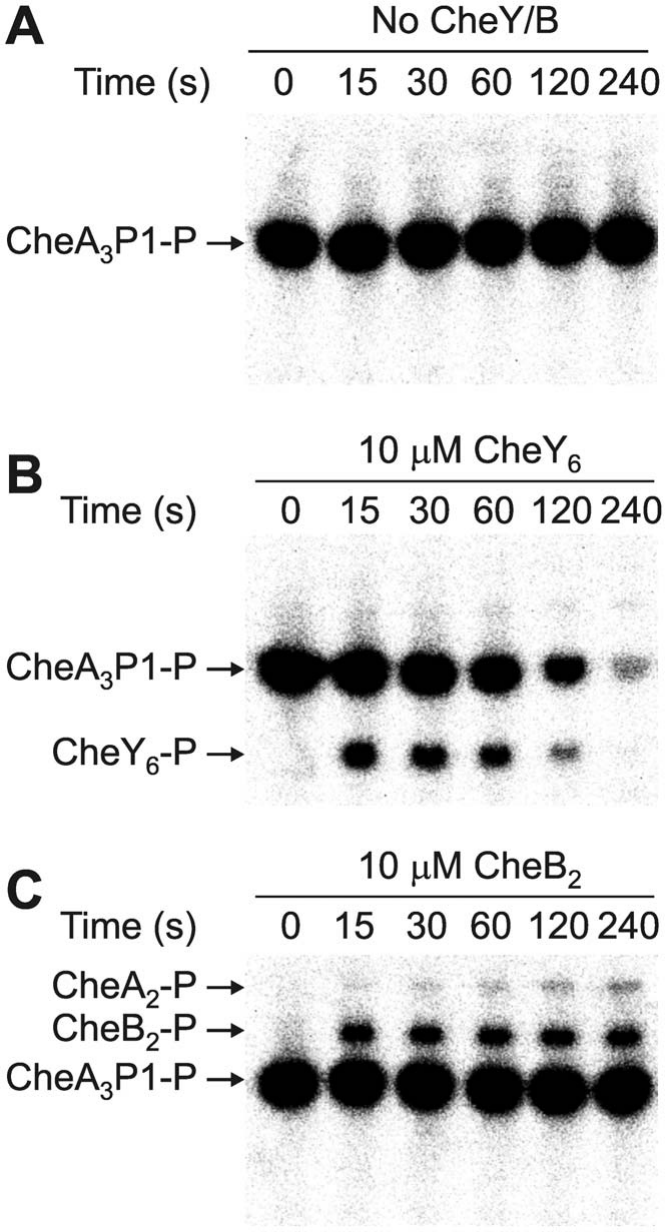
Phosphorimages of SDS-PAGE gels measuring phosphotransfer rate from CheY_6_-P and CheB_2_-P to CheA_2_. 30 µM CheA_3_P1-^32^P was incubated with 5 µM CheA_2_ for 1 hour prior to the addition of (A) reaction buffer, (B) 10 µM CheY_6_ and (C) 10 µM CheB_2_. 10 µl reaction samples were then taken at the time points indicated and quenched in 20 µl of 1.5 X SDS/EDTA loading dye. The quenched samples were analyzed by SDS-PAGE and detected by phosphorimaging.

The parameters for the phosphotransfer reactions were obtained by parameter fitting the previously published *R. sphaeroides* chemotaxis phosphotransfer assay data [43], [45], [48], where CheA-^32^P served as a phosphodonor to the RRs (Table 3). In the few cases where the assays were not very sensitive to the rate of reversed phosphotransfer from RR-P to CheA, reliable estimates of these rates were obtained using alternative phosphotransfer assays, in which RR-P was mixed with unphosphorylated CheA_2_ or CheA_3_ (examples shown in Figure 2). In these reactions, RR-P was generated using purified phosphorylated CheA P1 domains (either CheA_2_P1-P or CheA_3_P1-P) as the phosphodonor; control reactions lacking RR showed no phosphotransfer from CheA_2_P1-P or CheA_3_P1-P to either CheA_2_ or CheA_3_. These experiments showed that while CheA_2_ is phosphorylated by CheB_2_-P (Figure 2C) it is not phosphorylated by CheY_6_-P (Figure 2B). The parameter values obtained from these phosphotransfer reactions were then used in constructing the model.

**Table 3:** Parameter values estimated indirectly by fitting to phosphotransfer reaction data.

Rate	Description	Value	Units	Source
*k_3_*	CheA_2P_ to CheY_3_ phosphotransfer	6.60×10^3^	(Ms)^−1^	This study using [45]
*k_−3_*	CheA_2P_ to CheY_3_ reverse phosphotransfer	1.17×10^4^	(Ms)^−1^	This study using [45]
*k_4_*	CheA_2P_ to CheY_4_ phosphotransfer	8.85×10^5^	(Ms)^−1^	This study using [45]
*k_−4_*	CheA_2P_ to CheY_4_ reverse phosphotransfer	2.32×10^5^	(Ms)^−1^	This study using [45]
*k_5_*	CheA_2P_ to CheY_6_ phosphotransfer	1.54×10^3^	(Ms)^−1^	This study using [45]
*k_−5_*	CheA_2P_ to CheY_6_ reverse phosphotransfer	0	(Ms)^−1^	This study
*k_6_*	CheA_2P_ to CheB_1_ phosphotransfer	1.78×10^6^	(Ms)^−1^	This study using [45]
*k_−6_*	CheA_2P_ to CheB_1_ reverse phosphotransfer	2.85×10^6^	(Ms)^−1^	This study using [45]
*k_7_*	CheA_2P_ to CheB_2_ phosphotransfer	3.07×10^3^	(Ms)^−1^	This study using [45]
*k_−7_*	CheA_2P_ to CheB_2_ reverse phosphotransfer	1.53×10^3^	(Ms)^−1^	This study
*k_8_*	CheA_3P_ to CheY_6_ phosphotransfer	7.75×10^5^	(Ms)^−1^	This study using [43]
*k_−8_*	CheA_3P_ to CheY_6_ reverse phosphotransfer	2.83×10^3^	(Ms)^−1^	This study using [43]
*k_9_*	CheA_3P_ to CheB_2_ phosphotransfer	6.15×10^4^	(Ms)^−1^	This study using [43]
*k_−9_*	CheA_3P_ to CheB_2_ reverse phosphotransfer	3.10×10^3^	(Ms)^−1^	This study using [43]

### Response regulator dephosphorylation rates show CheY_6_ acts as a phosphate sink

*R. sphaeroides* responds to brief stimuli, returning to prestimulus behavior in less than 1 s [50]. This requires a rapid rate of signal termination. The measured autodephosphorylation half-times of the chemotaxis RRs, however, vary from ∼4 s for CheY_6_-P to ∼4000 s for CheB_1_-P (Table 4). As *R. sphaeroides* does not have a CheZ homologue, an alternative dephosphorylation mechanism is required. Recently, CheA_3_ was shown to be a specific phosphatase for CheY_6_-P [48], but no phosphatases have been identified for the remaining chemotaxis RRs.

**Table 4:** Comparison of RR-P autodephosphorylation rates with the RR-P dephosphorylation half-times predicted by the model.

		Dephosphorylation half-time predicted by simulation (s)†						
	Autodephosphorylation half-time (s)*	Wild type cells	Δ*cheY_3_*	Δ*cheY_4_*	Δ*cheY_6_*	Δ*cheB_1_*	Δ*cheB_2_*	No phosphatase‡
CheY_3_-P	36±3	4.9	n/a	4.7	296	3.7	5.2	8.4
CheY_4_-P	38±3	7.3	7.3	n/a	543	5.1	8.1	15.1
CheY_6_-P	4.1±0.3	1.3	1.3	1.4	n/a	1.3	1.3	8.2
CheB_1_-P	4046±150	4.2	4.2	4.0	309	n/a	4.5	8.1
CheB_2_-P	52±4	4.8	4.9	4.8	430	4.1	n/a	6.3

*: These values were calculated from the experimentally determined *in vitro* autodephosphorylation rate constants [48].

**†:** The model was allowed to reach a steady state where CheA_2_ autophosphorylation (reaction 1) and the phosphorylation of CheA_3_ by CheA_4_ (reaction 2) were both active. Then reactions 1 and 2 were turned off. These half-times represent the time taken for levels of each of the RR-Ps to fall to half of their steady state values. Deletion of RRs was simulated by setting their total concentration in the model to zero, e.g. for Δ*cheY_3_*, *Y_3T_* = 0 (Table 2).

**‡:** Lack of CheA_3_ phosphatase activity was simulated by setting *k*_15*a*_ = *k*_15*b*_ = 0 (Table 2).

Phosphate sinks have been shown to be involved in signal termination in several bacterial signaling pathways [51]–[54]. To test whether a similar mechanism operates in *R. sphaeroides*, we used the model to predict the decay timecourse of RR-P levels resulting from simultaneously switching off autophosphorylation of CheA_2_ (reaction 1 in Table 1) and the phosphorylation of CheA_3_ by CheA_4_ (reaction 2 in Table 1). Although, the model incorporates the experimentally determined autodephosphorylation rates (reactions 10–15b of Table 1), interestingly, the model predicts that levels of all of the RR-Ps decay with half-lives shorter than ∼7 s (Table 4), which is much faster than their experimentally measured autodephosphorylation rates. Within the model, only CheY_6_-P has a phosphatase. The only route by which the model could predict dephosphorylation rates for the other RR-Ps that are faster than their autodephosphorylation rates is for one or more of the RRs to be acting as “phosphate sinks”, with the dephosphorylation of the target RR-P proceeding via reversed phosphotransfer to a CheA, which in turn transfers the phosphoryl group to the sink RR.

To determine which RRs could act as sinks, we simulated RR-P decay rates in cells deleted for a single RR e.g. for the cell lacking CheY_3_ we changed *Y_3T_* to zero and measured the simulation half-lives of the remaining RR-Ps. We found that only the removal of CheY_6_ greatly increased the simulation half-lives of the remaining RR-Ps (Table 4), suggesting that CheY_6_ acts as a phosphate sink for all of the other RR-Ps. Without CheY_6_, the simulation half-lives of the remaining RRs were in some cases (CheY_3_-P, CheY_4_-P and CheB_2_-P) increased beyond their autodephosphorylation half-times (Table 4). This is the result of significant quantities of CheA_3_-P and CheA_2_-P being present at steady state, and persisting for some time after the autophosphorylation reactions (reactions 1 and 2) were turned off. This allows levels of RR-P to be replenished resulting in a RR-P simulation half-time that is slower than their autodephosphorylation half-times. Interestingly, even in the absence of CheY_6_, the simulated dephosphorylation rate of CheB_1_-P was faster than its autodephosphorylation rate. In the absence of CheY_6_, the other RRs (CheY_3_, CheY_4_ and CheB_2_) may act as phosphate sinks for CheB_1_-P i.e. CheB_1_-P acts as a phosphodonor for CheA_2_ which in turn donates the phosphoryl groups to CheY_3_, CheY_4_ and CheB_2_. To test this we set the rate of phosphotransfer from CheB_1_-P to CheA_2_ (*k_−6_*) to zero and found that the simulated dephosphorylation half-time for CheB_1_-P was increased to 4295 s, which is comparable with its autodephosphorylation half-time indicating that in the absence of CheY_6_, one or more of other RRs therefore act as phosphate sinks for CheB_1_.

To confirm that the ability of CheY_6_ to act as a phosphate sink was robust to changes in parameters we performed a sensitivity analysis, where we varied each parameter by factors of 0.1, 0.5, 1.5 and 10, and measured the effect on the simulation half-life of CheB_1_-P (Table S1). For the parameters that were determined experimentally, the standard error lies well within the range covered by factors of 0.5 and 1.5. The simulation half-life of CheB_1_-P was robust to large changes in the majority of parameters, and in all cases remained much faster than the CheB_1_-P autodephosphorylation rate, but as would be expected showed some sensitivity towards those parameters directly involved in the operation of the CheY_6_ phosphate sink i.e. the rates of phosphotransfer between CheB_1_ and CheA_2_ and between CheA_2_ and CheY_6_. In addition, the system was also sensitive to large changes, well outside the range covered by experimental error in parameter determination, in the total concentrations of CheA_2_ and CheY_6_. This sensitivity analysis indicates that the parameter space in which the phosphate sink mechanism will work efficiently is broad and extends well beyond the range of experimental errors in the parameters themselves, suggesting that this pathway is likely to operate *in vivo*.

**Figure 3:**
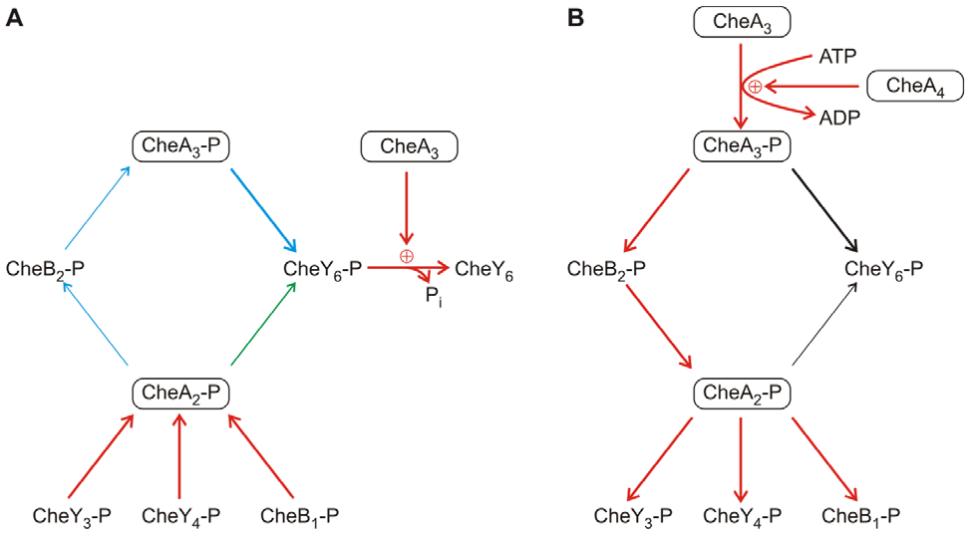
Summary of the phosphate-sink and phosphorelay pathways. (A) The phosphotransfer reactions that allow CheY_6_ to work as a phosphate-sink for the other RR-Ps. CheY_3_-P, CheY_4_-P, CheB_1_-P can act as phosphodonors for CheA_2_. Phosphoryl groups can take either of two routes from CheA_2_ to the CheY_6_-phosphate-sink. CheA_2_-P can directly phosphorylate CheY_6_ (shown in green) or alternatively, can phosphorylate CheB_2_, which can transfer the phosphoryl group to CheA_3_ which then phosphorylates CheY_6_ (shown in blue). CheY_6_-P is rapidly dephosphorylated due to the specific phosphatase activity of the bifunctional enzyme CheA_3_. For diagrammatic simplicity, reversible reactions are shown as operating in the direction that leads towards the sink. (B) Diagram summarizing the role of CheB_2_ and CheA_2_ in relaying phosphoryl groups from CheA_3_-P to its non-cognate RRs, CheY_3_, CheY_4_ and CheB_1_. The reactions necessary for this phosphorelay are highlighted in red. At the cytoplasmic chemotaxis cluster, phosphoryl groups are transferred from CheA_3_-P to CheY_6_ and CheB_2_. CheB_2_-P then diffuses to the polar chemotaxis cluster where it serves as a phosphodonor for CheA_2_. CheA_2_-P subsequently acts as a phosphodonor for CheY_3_, CheY_4_ and CheB_1_. For diagrammatic simplicity, reactions which are reversible are shown as operating in the direction that leads towards the non-cognate RRs of CheA_3_.

In summary, these simulated data suggest that not only is CheY_6_ a key regulator of flagellar motor rotation in *R. sphaeroides*, but it also acts as a “phosphate sink” ensuring rapid dephosphorylation of the other chemotaxis RRs (Figure 3A). This is very different from the *S. meliloti* sink where the sink CheY does not bind to the flagellar motor.

### The phosphatase activity of CheA_3_ is required for CheY_6_ to work as an efficient phosphate sink

In addition to containing the Hpt domain needed for phosphorylation of CheY_6_, CheA_3_ is also a phosphatase specific for CheY_6_-P. This phosphatase activity has previously been shown to be essential for chemotaxis [48]. We used the model to determine the effect of phosphatase removal on RR-P levels and signal termination times, by setting the rate constants for the CheA_3_ phosphatase reactions (15a) and (15b) in Table 2 to zero. The model predicted very high steady state concentrations of all of the RR-Ps (Table 5), with phosphorylation levels of the total chemotaxis RR pool rising from ∼55% to ∼97%. This was the result of increased levels of CheY_6_-P (due to decreased dephosphorylation) leading to higher CheA_2_-P and CheA_3_-P concentrations and therefore higher levels of the other RR-Ps. The model also predicted that the signal termination times for all of the RR-Ps would be longer without the phosphatase (Table 4), as CheY_6_ would be less effective as a phosphate sink for the other RR-Ps. The model therefore highlights the importance of the phosphatase activity in CheA_3_, and demonstrates that although it is specific for CheY_6_-P, the phosphatase activity indirectly affects the concentration of the other RR-Ps and their signal termination rates as outlined above (Figure 3A). Removal of the phosphatase activity is therefore predicted to cause a general increase in RR-P levels, which could account for the non-chemotactic phenotype of the strains lacking phosphatase activity [48].

**Table 5:** Comparison of steady state levels of RR-P with and without CheA_3_ phosphatase activity.

	Fraction phosphorylated (%)	
Protein	Wild-type cells	No phosphatase‡
CheY_3_	30	88
CheY_4_	75	98
CheY_6_	64	99
CheB_1_	33	91
CheB_2_	40	96
Total RR pool	55	97

**‡:** Lack of CheA_3_ phosphatase activity was simulated by setting *k*_15*a*_ = *k*_15*b*_ = 0 (Table 2).

### A phosphorelay pathway connects both chemotaxis clusters

**Figure 4:**
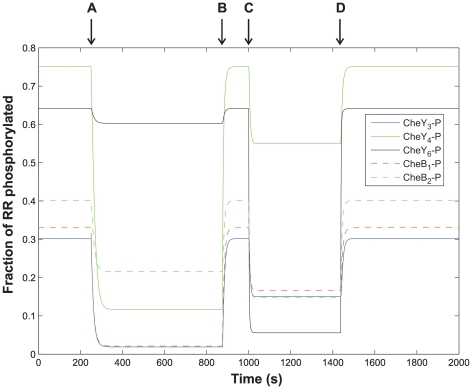
The predicted variation in levels of RR-P throughout a simulated chemotaxis response. Initially, CheA_2_ autophosphorylation in the polar cluster and the phosphorylation of CheA_3_ by CheA_4_ in the cytoplasmic cluster are occurring. To mimic attractant stimulation of the polar cluster, CheA_2_ autophosphorylation is turned off (*k_1_* = 0) at the point labelled (A). This causes a drop in RR-P levels and a new steady state is reached. (B) Subsequently, CheA_2_ autophosphorylation is turned back on and the system returns to its original steady state. At point (C), the phosphorylation of CheA_3_ by CheA_4_ is turned off (*k_2_* = 0) to mimic attractant stimulation of the cytoplasmic cluster. As a result of this, RR-P levels fall and a new steady state is reached. (D) Finally, phosphorylation of CheA_3_ by CheA_4_ is turned back on and the system returns to its initial steady state.

We modeled the consequences of chemoeffector stimulation of either of the two chemotaxis clusters by either (i) turning off CheA_2_ autophosphorylation (reaction (1) – parameter *k*_1_ set to zero) to mimic attractant stimulation of the polar chemotaxis cluster or (ii) turning off the phosphorylation of CheA_3_ by CheA_4_ (reaction (2) – parameter *k*_2_ set to zero) to mimic attractant stimulation of the cytoplasmic chemotaxis cluster (Figure 4). As expected, when CheA_2_ autophosphorylation was turned off (case (i)) there was a reduction in the phosphorylation levels of each of the RRs (Figure 4) because CheA_2_-P serves as a phosphodonor for all of the RRs. However, counter-intuitively, significant levels of all RR-Ps remained, including CheY_3_-P, CheY_4_-P and CheB_1_-P, which can only be generated by CheA_2_-P.

Analysis of the modeling results from case (i) revealed that even though CheA_2_ autophosphorylation had been turned off, significant levels of CheA_2_-P were present and were being generated by the reversal of reaction (7) (Table 1) i.e. CheB_2_-P was acting as a phosphodonor for CheA_2_ (as demonstrated in Figure 2C). In this case, CheY_6_ and CheB_2_ were phosphorylated by CheA_3_-P; CheB_2_-P then served as a phosphodonor for CheA_2_ i.e. CheB_2_ transfers phosphoryl groups between CheA_3_-P and CheA_2_. The CheA_2_-P generated in this way could then phosphorylate CheY_3_, CheY_4_ and CheB_1_. This result suggests that CheA_3_-P is linked to the RRs, CheY_3_, CheY_4_ and CheB_1_, via a multistep phosphorelay i.e. CheA_3_-P (His) to CheB_2_ (Asp) to CheA_2_ (His) to CheY_3_/CheY_4_/CheB_1_ (Asp).

In case (ii), switching off the phosphorylation of CheA_3_ by CheA_4_ caused a reduction not only in the levels of CheY_6_-P and CheB_2_-P, but also in levels of CheY_3_-P, CheY_4_-P and CheB_1_-P. Levels of CheY_3_-P, CheY_4_-P and CheB_1_-P were affected because (a) they were dephosphorylated faster since the reduction in CheY_6_-P levels was accompanied by an increase in the unphosphorylated CheY_6_ levels which acts as a phosphate sink and (b) there was no input of phosphoryl groups at the cytoplasmic cluster for CheB_2_ to transfer to these RRs via CheA_2_. These results indicate that even when CheA_2_ autophosphorylation is occurring, the bifunctional kinase/phosphatase in the cytoplasmic chemotaxis cluster makes a significant contribution to the phosphorylation levels of all of the RRs.

We performed a sensitivity analysis to look at the effect of varying each of the model parameters on levels of CheY_4_-P when CheA_2_ autophosphorylation is turned off; under these conditions CheY_4_-P levels give a measure of the extent to which the phosphorelay is occurring (Table S2). The system was robust to changes in many of the parameters although as would be expected was sensitive to changes in parameters that directly affect either i) the rate of entry or exit of phosphoryl groups from the system e.g. rate of phosphorylation of CheA_3_ by CheA_4_, rate of CheY_6_-P dephosphorylation (autodephosphorylation and phosphatase assisted), and the expression levels of CheA_3_ and CheY_6_ or ii) the functioning of the phosphorelay e.g. the expression levels of CheA_2_ and CheB_2_, rates of phosphotransfer between CheA_3_ and CheB_2_, between CheB_2_ and CheA_2_, and between CheA_2_ and CheY_4_. However, despite this sensitivity in almost all cases at least some phosphorylation of CheY_4_ was predicted indicating that the phosphorelay remained operational. In the two remaining extreme cases, where CheY_6_ expression levels were ten times higher than usual or where the rate of phosphorylation of CheA_3_ by CheA_4_ was ten-fold lower than the measured rate, levels of all RR-Ps, not just CheY_4_-P, were extremely low. These results indicate that the phosphorelay operates over a broad range of parameter space, although the extent to which it operates is sensitive to large changes in some of the parameters.

## Discussion

The experimental work leading up to this study produced an outline architecture of the complex signaling network controlling *R. sphaeroides* chemotaxis [30]. However, the mechanism of integrating the signals produced by each of the signaling clusters to control the flagellar motor was unclear. Mathematical modeling has provided considerable insight into the probable functioning of simpler chemotaxis pathways [2], [5], [55], and a control engineering approach has recently been used in *R. sphaeroides* to discriminate between several possible mechanisms of CheY control of the flagellar motor [56]. In this study, a mathematical model of *R. sphaeroides* chemotaxis was formulated that integrates *in vivo* and *in vitro* biochemical data on the kinetic preferences of the signaling reactions with *in vivo* measurements of protein copy number. Analysis of the model revealed two interesting features of the signaling network, both of which rely on reversed phosphotransfer from RR-Ps to CheA. Firstly, rapid signal termination for all chemotaxis RR-Ps may be achieved by CheY_6_ acting as a phosphate sink in addition to being the primary motor control protein (Figure 3A). Secondly, a novel phosphorelay involving CheB_2_ appears to link the cytoplasmic and polar chemotaxis clusters (Figure 3B). Together these two network features provide the bifunctional kinase/phosphatase, CheA_3_, with the means to increase or decrease the concentration of all of the chemotaxis RR-Ps and therefore to regulate the output of the two chemosensory clusters.

### CheY_6_ is a phosphate sink for all of the chemotaxis RR-Ps

Phosphate sinks provide an alternative mechanism for dephosphorylating RR-Ps [51], [52], instead of simply hydrolyzing the phosphoryl group (as in autodephosphorylation or phosphatase-assisted-dephosphorylation), the phosphoryl group is transferred to a HPK, which subsequently transfers it to the “phosphate sink” RR. The phosphoryl group is then hydrolyzed from the “phosphate sink” by either autodephosphorylation or phosphatase-assisted-dephosphorylation. The model presented in this study predicted that signal termination occurs rapidly in *R. sphaeroides*, with all RR-Ps dephosphorylating with half-times of less than ∼7 s (Table 4). This is consistent with the observed stimulus response time of 1 s for *R. sphaeroides* [50], since with a decay half-time of ∼7 s, CheY-P levels could fall by ∼10% during 1s, which, assuming that the *R. sphaeroides* motor is as ultrasensitive to changes in CheY-P levels as the *E. coli* motor [57], would be sufficient to give a significant change in motor rotation bias.

Prior to this study, it was known that CheY_6_-P with the aid of its specific phosphatase, the bifunctional protein CheA_3_, could dephosphorylate rapidly [48], however, the other RR-Ps were known to autodephosphorylate with half-times in excess of 36 s, with CheB_1_-P taking over 4000 s. By removing each of the RRs in turn from the model, we found that CheY_6_ was acting as a “phosphate sink” for the other RR-Ps, since cells lacking CheY_6_ showed much slower dephosphorylation rates for the remaining RR-Ps (Table 4 and Figure 3A). Furthermore, we showed that removal of the phosphatase activity of CheA_3_ from the model increased the dephosphorylation half-times of all of the RR-Ps, indicating that the phosphatase activity is required for efficient operation of the CheY_6_ phosphate sink and rapid signal termination. This phosphate sink role for CheY_6_ is additional to its primary role as a direct regulator of flagellar rotation [39].

CheY_6_ differs in several ways from the prototypical phosphate sink, CheY1 from *S. meliloti* [51]. CheY_6_ directly controls flagellar motor rotation by binding FliM [39], [49], and has a dedicated phosphatase, in contrast, *S. meliloti* CheY1 does not bind FliM and appears to function only as a “phosphate sink”. Another fundamental difference is the rate of dephosphorylation; CheY_6_-P dephosphorylates much faster than the autodephosphorylation rates of the RRs for which it acts as a sink whereas *S. meliloti* CheY1-P does not autodephosphorylate any faster than the motor binding RR, CheY2-P, for which it is a sink. The *S. meliloti* sink does not need to dephosphorylate quickly because it does not directly affect flagellar rotation and so phosphoryl groups can be stored on it until autodephosphorylation occurs, in contrast, *R. sphaeroides* CheY_6_ is a key regulator of flagellar rotation and therefore requires rapid signal termination.

### A CheB_2_ mediated phosphorelay connects the polar and cytoplasmic signaling clusters

As part of our interrogation of the model, we simulated attractant stimulation of the polar chemotaxis cluster by turning off autophosphorylation of CheA_2_, while allowing phosphorylation of CheA_3_ by CheA_4_ to occur (Figure 4). Interestingly under these conditions, even though CheA_3_-P cannot directly phosphorylate CheY_3_, CheY_4_, and CheB_1_, the model predicted non-zero concentrations of these RR-Ps. This is the result of the action of a phosphorelay where phosphoryl groups from CheA_3_-P (His) are transferred to CheB_2_ (Asp) then to CheA_2_ (His) and subsequently to either CheY_3_, CheY_4_ or CheB_1_ (Asp) (Figure 3B). Direct testing of the *in vivo* importance of this phosphorelay is confounded by the dual role of CheB_2_, firstly as a chemoreceptor methylesterase and secondly as a potential intermediate in the phosphorelay. The methylesterase activity of CheB_2_ is required for normal chemotaxis and it is not possible to block the phosphorylation of CheB_2_ by mutagenesis without impairing the control of this methylesterase activity. It is therefore not known the extent to which this CheB_2_ mediated phosphorelay operates *in vivo*; however, the model does incorporate both *in vitro* kinetic preference data and *in vivo* protein expression levels, and this suggests that the phosphorelay may operate *in vivo*, allowing the cytoplasmic cluster to make a significant contribution to phosphorylation levels of the non-cognate RRs; CheY_3_, CheY_4_ and CheB_1_.

The model in this study does not include adaptation as this is a poorly understood process in *R. sphaeroides*, with little experimental data. However, it is possible that the adaptation pathway could act to reduce the elevated RR-P levels caused by a constant influx of phosphoryl groups to the polar cluster from the cytoplasmic cluster by modifying the polar receptors in such a way as to reduce the autophosphorylation rate of the polar kinase, CheA_2_ i.e. the cell could adapt to constant signals from the cytoplasmic cluster. Although when cells are performing chemotaxis and swimming through gradients of chemoeffector, signals from the cytoplasmic cluster will vary over time and will make a significant contribution to RR-P levels. The relative contribution of this phosphorelay to levels of CheY_3_-P, CheY_4_-P and CheB_1_-P will be modulated by signals coming through the transmembrane chemoreceptors that directly control the rate of CheA_2_ autophosphorylation and would be greatest when the autophosphorylation rate of CheA_2_ is low and the rate of phosphorylation of CheA_3_ by CheA_4_ is high. These conditions could arise when cells are swimming up a gradient of a specific attractant which is sensed by the transmembrane chemoreceptors, while the metabolic state of the cell is worsening due to, for example, decreasing concentrations of an essential nutrient (for which there may not be a transmembrane chemoreceptor). Under such conditions, the increased rate of phosphorylation of CheA_3_ by CheA_4_, coupled with the CheB_2_/CheA_2_ mediated phosphorelay, could raise levels CheY_3_-P, CheY_4_-P and CheY_6_-P, allowing cells to override their favourable response to the extracellular attractant and swim away from these unfavourable environments.

Numerous examples of other two-component systems employing phosphorelays have been described [58]–[61]; however, to the best of our knowledge this is the first example of a phosphorelay involving two distinct HPKs localized to different regions of the cell, and also, the first phosphorelay to be found in a chemotaxis signaling pathway. Given that over 50% of bacteria with any *che* genes have more than two *cheA*s [31], [32], [48], it seems likely that phosphorelays allowing communication between different CheA homologues could be involved in chemotactic signaling in a wide range of bacterial species.

### Signal integration by the cytoplasmic cluster

The polar cluster senses extracellular signals while the cytoplasmic cluster is believed to sense the metabolic state of the cell [30]. The concentration of each RR-P depends not only on polar kinase activity but also on the balance of kinase and phosphatase activity in the cytoplasmic cluster. The kinase activity of the cytoplasmic cluster resides in CheA_4_ and the phosphatase activity resides in CheA_3_; both proteins have P5 (regulatory) domains and it is therefore likely that both activities will be regulated by environmental stimuli [48], [62]. A stimulus that increases phosphatase activity would have the effect of reducing levels of all RR-Ps, since the action of the phosphatase would directly accelerate CheY_6_-P dephosphorylation leaving more unphosphorylated CheY_6_ to function as a phosphate sink for the other RR-Ps. Such a mechanism could allow the cytoplasmic cluster to tune or modulate signals coming from the polar cluster since increased phosphatase activity would lead to a general decrease in chemotaxis RR-P levels. In contrast, a stimulus that increased kinase activity at the cytoplasmic cluster would increase levels of all RR-Ps because CheA_3_-P would phosphorylate CheY_6_ and CheB_2_ directly; CheB_2_ would then shuttle the phosphoryl groups to CheA_2_ and from there onto the other RRs while phosphorylation of CheY_6_ would reduce its capacity as a phosphate sink resulting in a general increase in RR-P levels. Therefore the overall sensory output of the pathway depends critically on the relative activity of CheA_3_ and CheA_4_, and potentially provides a mechanism for signals about the metabolic state of the cell to modulate signals regarding the extracellular environment.

## Materials and Methods

### Mathematical model

Full details on the mathematical model are included in Text S1. Briefly, the law of mass action was applied to the reactions detailed in Table 1 to produce a system of non-linear ordinary differential equations (ODEs), which were solved using Matlab (MathWorks). The model was parameterized with data from the literature [39], [43], [45], [48], [63]–[65] and our own experiments as detailed in Tables 2&3. A parameter fit of the phosphotransfer rates between each kinase and RR (time course data) with that of a mathematical model describing the *in vitro* reactions was performed. A number of local, global and genetic algorithm optimisation procedures were employed (simulated annealing, Hooke and Jeeves, least squares, Levenberg-Marquardt, Nelder-Mead, steepest descent and the genetic algorithm) to obtain a robust set of parameters (Table 3).

### Plasmids and strains

The plasmids and strains used are shown in Table S3. *E. coli* strains were grown in LB medium at 37°C. Where required, antibiotics were used at concentrations of 100 µg ml^−1^ for ampicillin and 25 µg ml^−1^ for kanamycin.

### Protein purification

His-tagged and GST-tagged *R. sphaeroides* CheA, CheY and CheB proteins were purified as described previously [66]. Protein purity and concentration was measured as described [45]. Purified proteins were stored at −20°C.

### Preparation of CheA_3_P1-^32^P

CheA_3_P1 was phosphorylated using [γ-^32^P] ATP and CheA_4_, and purified as described previously [48]. The final preparation of CheA_3_P1-^32^P was free of ATP and CheA_4_.

### Detection of phosphotransfer from the response regulators to CheA_2_

Assays were performed at 20°C in TGMNKD buffer (50 mM Tris HCl, 10% (v/v) glycerol, 5 mM MgCl_2_, 150 mM NaCl, 50 mM KCl, 1 mM DTT, pH 8.0). CheA_3_P1-^32^P was used to phosphorylate the RRs, CheY_6_ and CheB_2_, in these assays because it is a good phosphodonor for these proteins and even after prolonged incubation (>1 hour) CheA_3_P1-^32^P does not act as a direct phosphodonor for CheA_2_ (Figure 3A); therefore any CheA_2_-P generated in these assays is due to phosphotransfer from RR-P to CheA_2_ rather than direct phosphotransfer from CheA_3_P1-P to CheA_2_. 30 µM CheA_3_P1-^32^P was mixed with 5 µM CheA_2_ prior to the addition of 10 µM RR. Following the addition of RR, reaction aliquots of 10 µl were taken at the indicated timepoints and quenched immediately in 5 µl of 3 X SDS-PAGE loading dye (7.5% (w/v) SDS, 90 mM EDTA, 37.5 mM Tris HCl, 37.5% glycerol, 3% (v/v) β-mercaptoethanol, pH 6.8). Quenched samples were analyzed using SDS-PAGE and phosphorimaging as described previously.

### Protein expression levels

Protein expression levels were measured in wild-type *R. sphaeroides* cells grown under microaerobic growth conditions using quantitative immunoblotting as described previously [65], [67]–[69].

## Supporting Information

Table S1 The effect of parameter variation on the simulation half-life of CheB_1_-P.
(0.09 MB PDF)

Table S2 The effect of parameter variation on the predicted levels of CheY_4_-P when CheA_2_ autophosphorylation is turned off (*k_1_* = 0).
(0.09 MB PDF)

Table S3 Plasmids and bacterial strains used in this study.
(0.01 MB PDF)

Text S1 Mathematical modeling.
(0.02 MB PDF)
