# Supplementary material for: Modeling Chemotaxis Reveals the Role of Reversed Phosphotransfer and a Bi-Functional Kinase-Phosphatase
Source: PLoS Comput Biol. 2010 Aug 19;6(8):e1000896. doi: 10.1371/journal.pcbi.1000896 (PMC2924250; doi:10.1371/journal.pcbi.1000896)
Supplement: Table S1 — The effect of parameter variation on the simulation half-life of CheB1-P. (0.09 MB PDF) [file pcbi.1000896.s001.pdf]

Table S1. The effect of parameter variation on the simulation half-life of $CheB_1$-P.

		**Fold increase in $CheB_1$-P half time due to change in parameter value***			
**Parameter**	**Reaction**	**0.1 x k_i**	**0.5 x k_i**	**1.5 x k_i**	**10 x k_i**
k_1	A2 → A2P	1.2	1.1	1.0	1.0
k_2	A3 → A3P	0.9	0.9	1.1	1.1
k_3	A2P + Y3 → A2 + Y3P	1.0	1.0	1.0	1.0
k_{-3}	A2P + Y3 ← A2 + Y3P	1.0	1.0	1.0	1.0
k_4	A2P + Y4 → A2 + Y4P	1.0	1.0	1.0	0.9
k_{-4}	A2P + Y4 ← A2 + Y4P	0.9	1.0	1.0	1.0
$\boldsymbol{k_5}$	**A2P + Y6 → A2 + Y6P**	**3.9**	1.6	0.8	**0.2**
$\boldsymbol{k_6}$	**A2P + B1 → A2 + B1P**	0.6	0.8	1.2	**2.7**
$\boldsymbol{k_{-6}}$	**A2P + B1 ← A2 + B1P**	**2.8**	1.3	0.9	0.6
k_7	A2P + B2 → A2 + B2P	1.1	1.0	1.0	0.8
k_{-7}	A2P + B2 ← A2 + B2P	1.0	1.0	1.0	1.1
k_8	A3P + Y6 → A3 + Y6P	1.1	1.0	1.0	1.0
k_{-8}	A3P + Y6 ← A3 + Y6P	1.0	1.0	1.0	1.0
k_9	A3P + B2 → A3 + B2P	1.0	1.0	1.0	1.0
k_{-9}	A3P + B2 ← A3 + B2P	1.1	1.1	1.0	0.9
k_{10}	Y3P → Y3	1.0	1.0	1.0	1.0
k_{11}	Y4P → Y4	1.0	1.0	1.0	0.9
k_{12}	Y6P → Y6	1.1	1.1	1.0	0.8
k_{13}	B1P → B1	1.0	1.0	1.0	1.0
k_{14}	B2P → B2	1.0	1.0	1.0	1.0
k_{15a}	Y6P + A3 → Y6 + A3	1.6	1.2	0.9	0.8
k_{15b}	Y6P + A3P → Y6 + A3P	1.0	1.0	1.0	1.0
$\boldsymbol{A_{2T}}$	**Total [$CheA_2$]**	**3.8**	1.4	0.9	0.5
A_{3T}	Total [$CheA_3$]	1.1	1.1	0.9	0.7
Y_{3T}	Total [$CheY_3$]	1.0	1.0	1.0	1.1
Y_{4T}	Total [$CheY_4$]	0.9	1.0	1.0	1.5
$\boldsymbol{Y_{6T}}$	**Total [$CheY_6$]**	**5.3**	1.8	0.7	**0.1**
$\boldsymbol{B_{1T}}$	**Total [$CheB_1$]**	0.7	0.9	1.1	**3.6**
B_{2T}	Total [$CheB_2$]	1.1	1.0	1.0	0.8

* Values in bold indicate where a ≥2.5 fold change (up or down) has occurred
