# Supplementary material for: Modeling Chemotaxis Reveals the Role of Reversed Phosphotransfer and a Bi-Functional Kinase-Phosphatase
Source: PLoS Comput Biol. 2010 Aug 19;6(8):e1000896. doi: 10.1371/journal.pcbi.1000896 (PMC2924250; doi:10.1371/journal.pcbi.1000896)
Supplement: Table S2 — The effect of parameter variation on the predicted levels of CheY4-P when CheA2 autophosphorylation is turned off (k1 = 0). (0.09 MB PDF) [file pcbi.1000896.s002.pdf]

Table S2. The effect of parameter variation on the predicted levels of CheY$_4$-P when CheA$_2$ autophosphorylation is turned off ($k_1 = 0$).

		Fold increase in CheY$_4$-P levels due to change in parameter value*			
**Parameter**	**Reaction**	**0.1 x k_i**	**0.5 x k_i**	**1.5 x k_i**	**10 x k_i**
$\boldsymbol{k_2}$	**A3 → A3P**	**0.0**	**0.2**	**3.6**	**8.0**
k_3	A2P + Y3 → A2 + Y3P	1.0	1.0	1.0	1.0
k_{-3}	A2P + Y3 ← A2 + Y3P	1.0	1.0	1.0	1.0
$\boldsymbol{k_4}$	**A2P + Y4 → A2 + Y4P**	**0.1**	0.5	1.4	**4.4**
$\boldsymbol{k_{-4}}$	**A2P + Y4 ← A2 + Y4P**	**4.4**	1.7	0.7	**0.1**
$\boldsymbol{k_5}$	**A2P + Y6 → A2 + Y6P**	**2.5**	1.5	0.8	**0.1**
k_6	A2P + B1 → A2 + B1P	1.0	1.0	1.0	1.0
k_{-6}	A2P + B1 ← A2 + B1P	1.0	1.0	1.0	1.0
$\boldsymbol{k_7}$	**A2P + B2 → A2 + B2P**	1.2	1.1	0.9	**0.4**
$\boldsymbol{k_{-7}}$	**A2P + B2 ← A2 + B2P**	**0.1**	0.6	1.3	**2.8**
$\boldsymbol{k_8}$	**A3P + Y6 → A3 + Y6P**	**2.6**	1.5	0.7	**0.1**
k_{-8}	A3P + Y6 ← A3 + Y6P	0.8	0.9	1.1	2.0
$\boldsymbol{k_9}$	**A3P + B2 → A3 + B2P**	**0.1**	0.6	1.3	**3.0**
$\boldsymbol{k_{-9}}$	**A3P + B2 ← A3 + B2P**	1.9	1.4	0.8	**0.2**
k_{10}	Y3P → Y3	1.0	1.0	1.0	1.0
k_{11}	Y4P → Y4	1.0	1.0	1.0	0.7
$\boldsymbol{k_{12}}$	**Y6P → Y6**	2.2	1.4	0.8	**0.3**
k_{13}	B1P → B1	1.0	1.0	1.0	1.0
k_{14}	B2P → B2	1.0	1.0	1.0	0.8
$\boldsymbol{k_{15a}}$	**Y6P + A3 → Y6 + A3**	**6.7**	**3.7**	0.6	**0.2**
k_{15b}	Y6P + A3P → Y6 + A3P	1.0	1.0	1.0	0.8
$\boldsymbol{A_{2T}}$	**Total [CheA$_2$]**	**0.2**	0.6	1.3	2.3
$\boldsymbol{A_{3T}}$	**Total [CheA$_3$]**	**0.1**	0.5	1.3	**2.5**
Y_{3T}	Total [CheY$_3$]	1.0	1.0	1.0	1.0
Y_{4T}	Total [CheY$_4$]	1.0	1.0	1.0	0.7
$\boldsymbol{Y_{6T}}$	**Total [CheY$_6$]**	**8.2**	**6.8**	**0.3**	**0.0**
B_{1T}	Total [CheB$_1$]	1.0	1.0	1.0	1.0
$\boldsymbol{B_{2T}}$	**Total [CheB$_2$]**	**0.1**	0.6	1.3	**2.8**

* Values in bold indicate where a ≥2.5 fold change (up or down) has occurred
