# Supplementary material for: Modeling Chemotaxis Reveals the Role of Reversed Phosphotransfer and a Bi-Functional Kinase-Phosphatase
Source: PLoS Comput Biol. 2010 Aug 19;6(8):e1000896. doi: 10.1371/journal.pcbi.1000896 (PMC2924250; doi:10.1371/journal.pcbi.1000896)
Supplement: Table S3 — Plasmids and bacterial strains used in this study. (0.01 MB PDF) [file pcbi.1000896.s003.pdf]

Table S3. Plasmids and bacterial strains used in this study

Strain/plasmid	Description	Source/reference
E. coli strains		
M15pREP4	Expression host containing pREP4; kanamycin resistant	Qiagen
XL1 Blue	General cloning strain and expression host. *lacI*^{q}; tetracycline resistant	Stratagene
Plasmids		
pGEX6P-1	IPTG inducible expression vector. Introduces a GST tag at the N terminus of the expressed protein. Confers ampicillin resistance	GE Life Sciences
pGEXA4	$CheA_4$ expression plasmid. pGEX6P-1 derivative	[1]
pREP4	Plasmid containing the *lacI*^{q} gene. Compatible with pQE30 and pQE60. Confers kanamycin resistance	Qiagen
pQE30	IPTG inducible expression vector. Introduces $RGS(H)_6$ at the N terminus of the expressed protein. Confers ampicillin resistance	Qiagen
pQE60	IPTG inducible expression vector. Introduces $RGS(H)_6$ at the C terminus of the expressed protein. Confers ampicillin resistance	Qiagen
pQEA2	$CheA_2$ expression plasmid. pQE30 derivative	[2]
pQE60A3P1	$CheA_3$P1 expression plasmid. pQE60 derivative	[1]
pQEY1	$CheY_1$ expression plasmid. pQE30 derivative	[2]
pQEY2	$CheY_2$ expression plasmid. pQE30 derivative	[2]
pQEY3	$CheY_3$ expression plasmid. pQE30 derivative	[2]
pQEY4	$CheY_4$ expression plasmid. pQE30 derivative	[2]
pQEY5	$CheY_5$ expression plasmid. pQE30 derivative	[3]
pQEY6	$CheY_6$ expression plasmid. pQE30 derivative	[4]
pQEB1	$CheB_1$ expression plasmid. pQE30 derivative	[5]
pQEB2	$CheB_2$ expression plasmid. pQE30 derivative	[4]

References

1. Porter SL, Roberts MAJ, Manning C.S., Armitage JP (2008) A bifunctional kinase-phosphatase in bacterial chemotaxis. Proc Natl Acad Sci USA 105: 18531-18536.

2. Shah DS, Porter SL, Harris DC, Wadhams GH, Hamblin PA, Armitage JP (2000) Identification of a fourth *cheY* gene in *Rhodobacter sphaeroides* and interspecies interaction within the bacterial chemotaxis signal transduction pathway. Mol Microbiol 35: 101-112.

3. Porter SL, Armitage JP (2002) Phosphotransfer in *Rhodobacter sphaeroides* chemotaxis. J Mol Biol 324: 35-45.

4. Porter SL, Warren AV, Martin AC, Armitage JP (2002) The third chemotaxis locus of *Rhodobacter sphaeroides* is essential for chemotaxis. Mol Microbiol 46: 1081-1094.

5. Martin AC, Wadhams GH, Shah DSH, Porter SL, Mantotta JC, Craig TJ, Verdult PH, Jones H, Armitage JP (2001) CheR- and CheB-dependent chemosensory adaptation system of *Rhodobacter sphaeroides*. J Bacteriol 183: 7135-7144.
