# Supplementary material for: Modeling Chemotaxis Reveals the Role of Reversed Phosphotransfer and a Bi-Functional Kinase-Phosphatase
Source: PLoS Comput Biol. 2010 Aug 19;6(8):e1000896. doi: 10.1371/journal.pcbi.1000896 (PMC2924250; doi:10.1371/journal.pcbi.1000896)
Supplement: Text S1 — Mathematical modeling. (0.02 MB PDF) [file pcbi.1000896.s004.pdf]

Text S1

Mathematical modeling

Our mathematical model consists of a system of coupled ordinary differential equations (ODEs) which describe the dynamic concentrations of the phosphotransfer proteins throughout the *R. sphaeroides* cell. We applied the law of mass action to the phosphotransfer reactions in Table 1 and obtained the following system of non-linear ODEs.

$$\frac{dA_2}{dt} = -k_1A_2 + k_3A_{2P}Y_3 - k_{-3}A_2Y_{3P} + k_4A_{2P}Y_4 - k_{-4}A_2Y_{4P} + k_5A_{2P}Y_6 - k_{-5}A_2Y_{6P} + k_6A_{2P}B_1 - k_{-6}A_2B_{1P} + k_7A_{2P}B_2 - k_{-7}A_2B_{2P} \quad (1)$$

$$\frac{dA_{2P}}{dt} = k_1A_2 - k_3A_{2P}Y_3 + k_{-3}A_2Y_{3P} - k_4A_{2P}Y_4 + k_{-4}A_2Y_{4P} - k_5A_{2P}Y_6 + k_{-5}A_2Y_{6P} - k_6A_{2P}B_1 + k_{-6}A_2B_{1P} - k_7A_{2P}B_2 + k_{-7}A_2B_{2P} \quad (2)$$

$$\frac{dA_3}{dt} = -k_2A_3 + k_8A_{3P}Y_6 - k_{-8}A_3Y_{6P} + k_9A_{3P}B_2 - k_{-9}A_3B_{2P} \quad (3)$$

$$\frac{dA_{3P}}{dt} = k_2A_3 - k_8A_{3P}Y_6 + k_{-8}A_3Y_{6P} - k_9A_{3P}B_2 + k_{-9}A_3B_{2P} \quad (4)$$

$$\frac{dY_3}{dt} = -k_3A_{2P}Y_3 + k_{-3}A_2Y_{3P} + k_{10}Y_{3P} \quad (5)$$

$$\frac{dY_{3P}}{dt} = k_3A_{2P}Y_3 - k_{-3}A_2Y_{3P} - k_{10}Y_{3P} \quad (6)$$

$$\frac{dY_4}{dt} = -k_4A_{2P}Y_4 + k_{-4}A_2Y_{4P} + k_{11}Y_{4P} \quad (7)$$

$$\frac{dY_{4P}}{dt} = k_4A_{2P}Y_4 - k_{-4}A_2Y_{4P} - k_{11}Y_{4P} \quad (8)$$

$$\frac{dY_6}{dt} = -k_5A_{2P}Y_6 + k_{-5}A_2Y_{6P} - k_8A_{3P}Y_6 + k_{-8}A_3Y_{6P} + k_{12}Y_{6P} + k_{15a}Y_{6P}A_3 + k_{15b}Y_{6P}A_{3P} \quad (9)$$

$$\frac{dY_{6P}}{dt} = k_5A_{2P}Y_6 - k_{-5}A_2Y_{6P} + k_8A_{3P}Y_6 - k_{-8}A_3Y_{6P} - k_{12}Y_{6P} - k_{15a}Y_{6P}A_3 - k_{15b}Y_{6P}A_{3P} \quad (10)$$

$$\frac{dB_1}{dt} = -k_6A_{2P}B_1 + k_{-6}A_2B_{1P} + k_{13}B_{1P} \quad (11)$$

$$\frac{dB_{1P}}{dt} = k_6A_{2P}B_1 - k_{-6}A_2B_{1P} - k_{13}B_{1P} \quad (12)$$

$$\frac{dB_2}{dt} = -k_7A_{2P}B_2 + k_{-7}A_2B_{2P} - k_9A_{3P}B_2 + k_{-9}A_3B_{2P} + k_{14}B_{2P} \quad (13)$$

$$\frac{dB_{2P}}{dt} = k_7A_{2P}B_2 - k_{-7}A_2B_{2P} + k_9A_{3P}B_2 - k_{-9}A_3B_{2P} - k_{14}B_{2P} \quad (14)$$

Here A_i=[CheA$_i$], A_{iP}=[CheA$_{iP}$], Y_j=[CheY$_j$], Y_{jP}=[CheY$_{jP}$] , B_k=[CheB$_k$] and Y_{kP}=[CheY$_{kP}$] where *i=[2,3], j=[3,4,6]* and *k=[1,2]* with the reaction rates as given in Table S2.

In order to close the system of equations we defined a set of initial conditions:

$$A_i(\mathbf{x},0) = A_{i0},\ \ A_{iP}(\mathbf{x},0) = 0,\ \ Y_j(\mathbf{x},0) = Y_{j0},\ Y_{jP}(\mathbf{x},0) = 0,$$
$$B_k(\mathbf{x},0) = B_{k0}\ \text{ and }\ B_{kP}(\mathbf{x},0) = 0 \qquad (15)$$

Here A_{i0} is the initial concentration of CheA$_i$, Y_{j0} the initial concentration of CheY$_j$ and B_{k0} the initial concentration of CheB$_k$. The model was populated with the experimental data in Table S2 and equations (1)-(14), along with the respective initial conditions in equation (15), were solved using an adaptive time stepping ODE solver in Matlab (ode15s) due to the stiffness of the problem.
